# Classifying molecular phenotypes of *G6PC* variants for pathogenic properties and to guide therapeutic development

**DOI:** 10.1002/jmd2.12215

**Published:** 2021-03-28

**Authors:** Kathleen L. Plona, Jean F. Eastman, Mitchell L. Drumm

**Affiliations:** ^1^ Genetics and Genome Sciences Department, School of Medicine Case Western Reserve University Cleveland Ohio USA

**Keywords:** G6PC, genotype‐phenotype, glucose‐6‐phosphatase, glycogen storage disease, GSD1a

## Abstract

Due to advances in sequencing technologies, identification of genetic variants is rapid. However, the functional consequences of most genomic variants remain unknown. Consequently, variants of uncertain significance (VUS) that appear in clinical DNA diagnostic reports lack sufficient data for interpretation. Algorithms exist to aid prediction of a variant's likelihood of pathogenicity, but these predictions usually lack empiric evidence. To examine the feasibility of generating functional evidence in vitro for a given variant's role in disease, a panel of 29 coding sequence variants in the *G6PC* gene was assessed. *G6PC* encodes glucose‐6 phosphatase enzyme, and reduction in its function causes the rare metabolic disease glycogen storage disease type 1a (GSD1a). Variants were heterologously expressed as fusion proteins in a hepatocyte‐derived cell line and examined for effects on steady‐state protein levels, biosynthetic processing, and intracellular distribution. The screen revealed variant effects on protein levels, N‐linked glycosylation status, and cellular distribution. Of the eight VUS tested, seven behaved similar to wild‐type protein while one VUS, p.Cys109Tyr, exhibited features consistent with pathogenicity for all molecular phenotypes assayed, including significantly reduced protein levels, alteration in protein glycosylation status, and abnormally diffuse protein localization pattern, and has recently been reported in a patient with GSD1a. Our results show that such a screen can add empiric evidence to existing databases to aid in diagnostics, and also provides further classification for molecular phenotypes that could be used in future therapeutic screening approaches for small molecule or gene editing strategies directed at specific variants.

**Synopsis**

Systematic analysis of variants of uncertain significance and known disease causing gene variants yields information on pathogenicity and potential variant‐specific routes of therapeutic intervention.

## 1 INTRODUCTION

Increasing use of DNA sequencing, paired with technologies such as small molecule screening and genome editing, is providing hope that gene‐specific therapies will exist for an increasing number of disorders. However, with the decreasing cost of DNA sequencing, variant interpretation has become the limiting factor in its clinical use.^1^, ^2^ In silico predictive programs like CADD, SIFT, and Polyphen use multiple factors to assess pathogenic potential of variants, including sequence conservation and homology, physiochemical similarity, and predicted structural changes.^3^, ^4^, ^5^, ^6^ Variants are annotated as a spectrum, from pathogenic to benign, and those variants lacking evidence for their role in disease are listed as variants of unknown or uncertain significance (VUS). Guidelines from american college of medical genetics suggest only utilizing results when multiple in silico predictors agree on a classification, which leaves up to 35% of variants unclassified.^7^, ^8^, ^9^ Functional, empiric data regarding putative disease‐causing variants could provide direct evidence of pathogenicity outside the capabilities of current in silico prediction programs to address these cases of uncertainty and conflicting interpretations. Molecular and biochemical characterization of variants' effects could not only have diagnostic value in establishing pathogenicity, they could also guide variant‐specific therapeutic development in the emerging era of personalized medicine.

The effects of genetic variants on protein function has proven to be an important aspect of therapeutic development and in vitro characterizations have allowed the development of drugs and their indications to move at a rapid pace. For example, in cystic fibrosis research variants have been classified due to their effect on the protein product, and drugs have been identified that increase activity,^10^ and correct misfolding,^11^ for specific variants. Initially clinical trials were required to obtain approval for drug use in each variant; however, clinical trials are inherently complicated for rare diseases with small patient populations.^12^ Recently FDA approval was granted based on in vitro research, which is accelerated by the variant classifications helping predict which variants will respond to particular drugs.^13^ This therapeutic success demonstrates the utility of heterologous variant analysis, and provides an example of how such data can be integrated and applied to genetic disease management.

As a model genetic disease for testing methods to improve variant interpretation, we investigated glycogen storage disease type 1a (GSD1a [MIM232200]), caused by variants in the glucose‐6‐phosphatase gene (*G6PC* [MIM613742]). The *G6PC* gene is a nine transmembrane‐domain protein expressed in the liver, kidney, and small intestine where it functions in the endoplasmic reticulum (ER) to cleave glucose‐6‐phosphate in the terminal step of glycogenolysis and gluconeogenesis.^14^, ^15^, ^16^, ^17^ The catalytic site for phosphohydrolase function has been homology mapped with other phosphatases identifying key catalytic residues at Arg83, His119, Arg170, and His176,^18^ and its biosynthesis includes N‐linked glycosylation at N96.^19^ While much of the cell biology of G6PC is understood and many disease‐causing variants have been cataloged,^16^, ^20^, ^21^, ^22^, ^23^, ^24^, ^25^ there still remain numerous VUS, and within the known disease‐causing variants the molecular cause of pathogenicity is unclear. At the time of writing, the ClinVar database listed 145 variants, 87 of which are in the coding sequence, and 26 of those listed as VUS. For some known pathogenic variants, genotype/phenotype correlations have been explored on a clinical level in attempt to elucidate the role of specific genotypes in patient outcomes and improve precision medicine; however, extensive heterogeneity and external factors complicated this evaulation.^26^ Here, we provide a uniform approach to classifying variants in the *G6PC* gene, in vitro, according to their impact on biosynthesis of a readily detectable fusion protein. The goal of this study was to use a controlled system to evaluate the molecular phenotypes of variants on multiple characteristics affecting protein function, such as total protein at steady state, post‐translational modification, and subcellular localization. The factors analyzed here provide valuable in vitro data for understanding the variant spectrum, and could be broadly applicable to other genetic disorders informing diagnostics, prognostics, and therapeutic development.

## 2 MATERIALS AND METHODS

### 2.1 Cell culture and transfection

HepG2 cells (ATCC HB‐8065), which are routinely used as a robust in vitro model for the liver in metabolic studies,^27^ were cultured using DMEM/F‐12 growth medium with 10% fetal bovine serum, 1% (10 000 U/mL) penicillin/streptomycin, and 1% (110 mg/L) l‐glutamine at 37°C with 5% CO_2_. Cells are passaged at 80% to 100% confluence using 0.25% Trypsin (wt/vol)‐0.53 nM ethylenediaminetetraacetic acid. For transfection, nearly confluent cells were passaged 24 hours prior, then transfected following the Lipofectamine 3000 (ThermoFisher #L3000001) protocol for 24 well plates using 500 ng of each plasmid and 1.5 μL Lipofectamine 3000 reagent per well. Transfection mixture was left on cells for 48 hours before imaging, and for 72 hours before protein harvest for western blot analysis.

### 2.2 Plasmid construction

The fusion protein approach has been used for this enzyme by others and have shown that N‐terminal fusions do not appear to disrupt enzyme stability or function,^17^, ^19^, ^28^ nor do C‐terminal fusions.^2^, ^12^ N‐terminal G6PC‐EFGP fusion plasmid base constructs were purchased from VectorBuilder. The base plasmids were G6PC‐EGFP fusion plasmid containing the G6PC coding sequence with stop codon (TAA) removed and fused to EGFP with a single glycine (GGA) linker (VB190719‐1039cgw pRP[Exp]‐Neo‐SV40>[G6PC]:EGFP), and an EGFP only control plasmid (VB170206‐1119ntc pRP[Exp]‐Neo‐Sv40>EGFP). These plasmids were altered with restriction enzyme cloning to replace the NeoR selectable marker with mCherry fluorophore between SacI and XhoI sites. Individual variants were introduced to the plasmids using GeneArt site directed mutagenesis reagents and protocol (ThermoFisher #A13282) and confirmed via Sanger sequencing. Additionally, an unmodified G6PC‐FLAG construct (VB190521‐1108jbf pRP[Exp]‐mCherry‐SV40>hG6PC[NM_000151.3]/FLAG) was used as the wild type (WT) construct in colocalization analysis.

### 2.3 Fixing and staining cells

For steady‐state protein expression imaging, HepG2 cells were transfected as described above in 24‐well, clear‐bottom, black‐walled plates. At 48 hours, cells were rinsed with 1x phosphate‐buffered saline (PBS) and fixed in 4% paraformaldehyde for 10 minutes. After three washes in 1x PBS, cells were stained with 1 μg/mL 4′,6‐diamidino‐2‐phenylindole (DAPI) nuclear stain for 5 minutes then stored at 4°C in 1x PBS. For colocalization analysis, the G6PC‐FLAG plasmid was fixed as described then permeabilized with 0.1% PBS‐TritonX for 10 minutes, blocked in 10% donkey serum at room temperature for 1 hour, incubated with mouse anti‐FLAG primary antibody diluted 1:500 in 10% donkey serum (Sigma #F1804, lot SLBX2256) at 4°C overnight, rinsed five times in 1x PBS, incubated with Donkey anti‐mouse‐Alexa 647 secondary antibody diluted 1:1000 in 10% donkey serum (Jackson ImmunoResearch #715‐605‐150) for 1 hour, rinsed five times, and stained with 1 μg/mL DAPI for 5 minutes followed by storage in 1x PBS at 4°C in the dark until imaging.

### 2.4 Operetta imaging and analysis

Cells were imaged and analyzed for steady‐state protein expression level on the Perkin Elmer Operetta System and Columbus software. Transfected cells fixed and DAPI stained on 24 well plates were imaged at ×20 magnification with 60 images taken per variant, in triplicate, for a total of n = 180 images analyzed per variant. Each image was analyzed for cell number via DAPI staining, transfected cell number via mCherry fluorescence, and G6PC‐expressing cell number via EGFP fluorescence. Output was given as percent of transfected (mCherry positive) cells expressing detectable levels of G6PC (EGFP positive) at a preset threshold determined from background levels in negative controls, and compared to WT using Welch's two‐tailed *t* tests for statistical significance.

### 2.5 Western blot

Cells were lysed in 10 mM HEPES pH 7.3 NaCl 1% NP‐40 with protease inhibitor (Millipore Sigma #4693132001). Collected protein was denatured at 37°C for 30 minutes without addition of 2‐mercaptoethanol and 20 μg total protein lysate per lane was run on western blot. Primary antibody was rat anti‐GFP (Biolegend #338002) at 1:2000 and secondary goat anti‐rat IgG horseradish peroxidase (HRP) conjugated (Millipore Sigma #AB183P) at 1:5000, both diluted in 5% milk in 1x PBS with 0.1% Tween20.

### 2.6 Deglycosylation

Cells were transfected as described above for 48 hours and 30 μg of total protein lysate per sample were deglycosylated with PNGase Fast Kit (Sigma‐Aldrich #EMS0001) following the manufacturer's protocol with the denaturing conditions modified to 50°C for 10 minutes. The entire treated sample was run on western blot as described above. Antibodies used for this blot were rat anti‐GFP (Biolegend #338002) at 1:2000, goat anti‐rat IgG HRP conjugated (Millipore Sigma #AP183P) at 1:5000, mouse anti‐mCherry (Novus Biologicals #NBP1‐96752) at 1:2000, goat anti‐mouse IgG HRP conjugated (Millipore Sigma #AP181) at 1:5000, rabbit anti‐vinculin (Abcam #ab129002) at 1:10 000, and mouse anti‐rabbit IgG HRP conjugated (Millipore Sigma #AP188P) at 1:5000.

### 2.7 High magnification imaging and colocalization analysis

HepG2 cells grown on 24‐well glass bottom plates were transfected, fixed, and stained as described above and imaged on the Zeiss Axio Observer 7 Scope with Zen Pro 3.0 Software (Carl Zeiss Microscopy, 2019, Germany). Each variant modeled on the G6PC‐EGFP (green) plasmid was cotransfected with a WT G6PC‐FLAG (far red) construct to assess colocalization. A plasmid expressing EGFP alone was used as a reference for cytoplasmic localization, and each plasmid independently expressed mCherry to allow identification of transfected cells (Figure 4A). Images were taken of nine cells per variant at ×63 magnification for analysis using the Zeiss colocalization module software and a Pearson correlation coefficient was reported for each individual cell.

## 3 RESULTS

**FIGURE 1:**
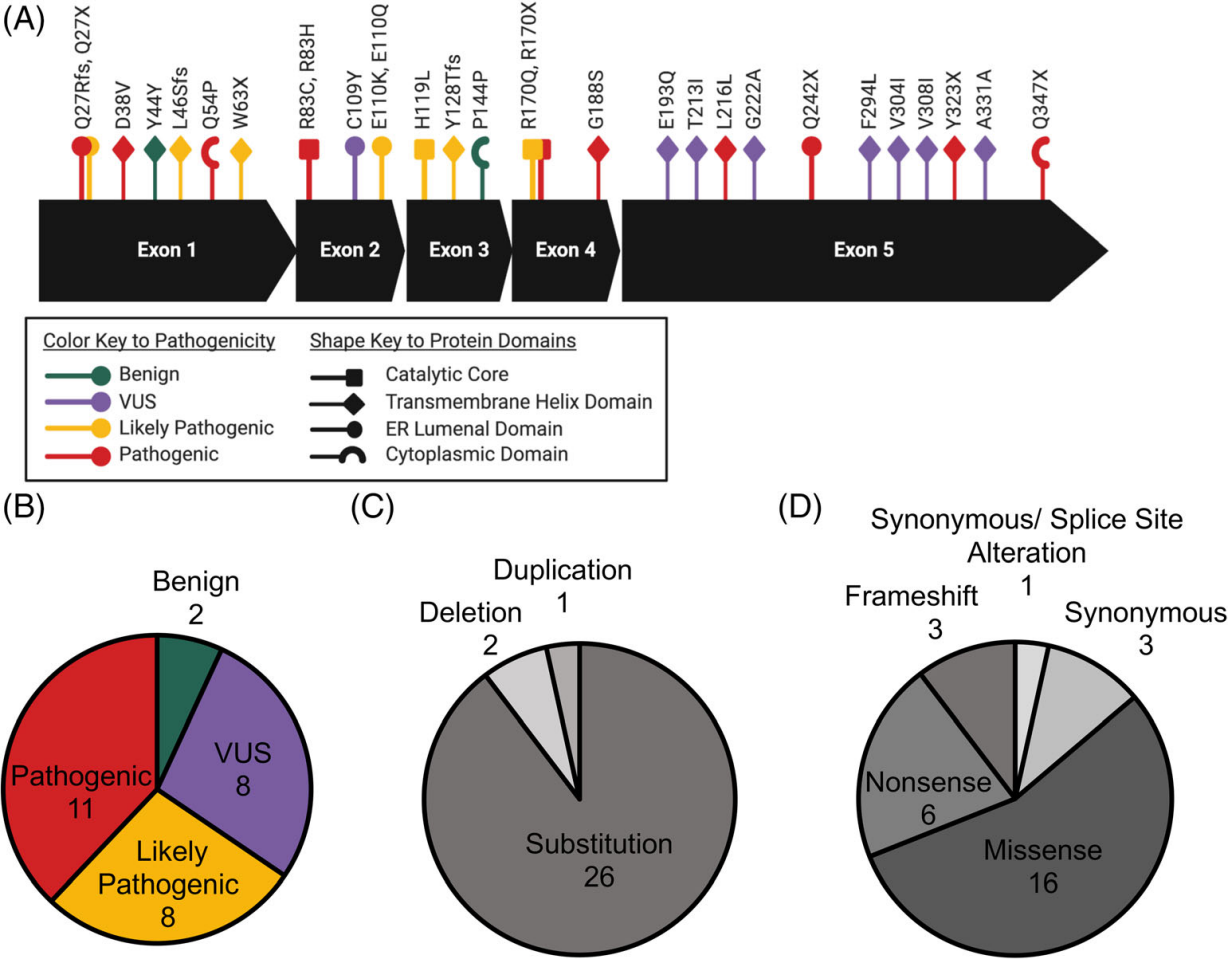
Selection of G6PC variants. Characteristics of selected G6PC variants. A, Schematic of G6PC coding sequence with select panel of 29 G6PC variants shown with reported pathogenicity (color) and protein domain location (shape). Number of variants represented from each type of (B) reported pathogenicity rating (compiled from ClinVar and Ensembl databases), (C) DNA sequence variant, and (D) protein variant

Fluorescent and epitope‐tagged G6PC fusion proteins were used to do large‐scale characterization of several aspects of G6PC including quantity, biosynthesis, and intracellular localization. In total, 29 variants spanning all five exons of *G6PC* (Figure 1A) were selected to represent a variety of pathogenicity ratings (Figure 1B), DNA alterations (Figure 1C), and protein alterations (Figure 1D). As the majority of reported variants in *G6PC* are substitutions, our panel reflects that by testing 26 substitutions, 2 deletions, and 1 duplication. Two known benign variants were included as controls along with a pathogenic variant, p.Lys216=, that affects splicing but not protein coding, and thus should be functionally benign in our model using the intronless cDNA sequence. The remaining 10 pathogenic variants were tested to further elucidate their molecular mechanisms of pathogenicity, while the properties of variants annotated as “likely pathogenic” and “VUS” helps in their classification as well.

**FIGURE 2:**
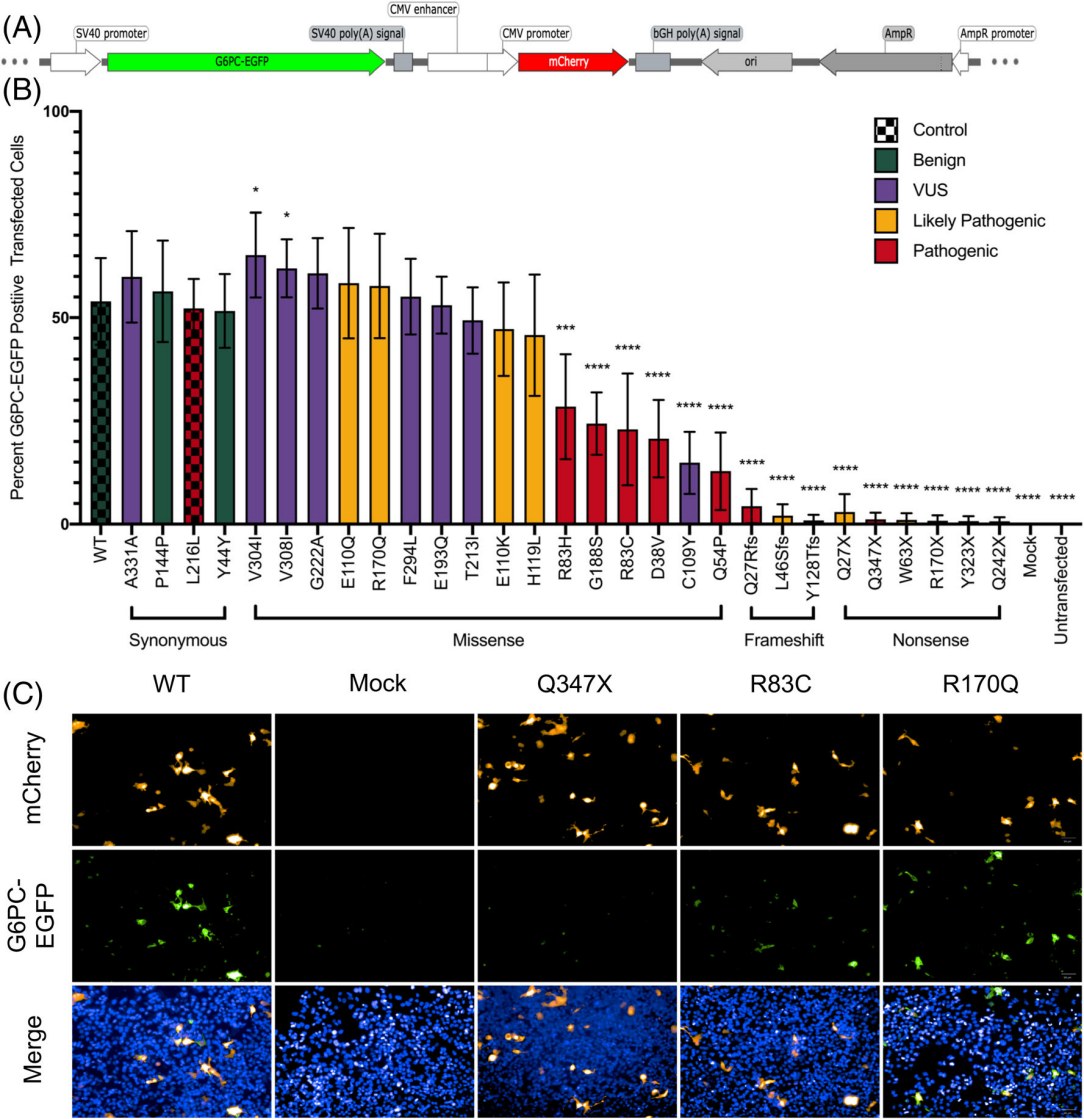
Protein levels are significantly reduced for a subset of G6PC variants. Protein expression of G6PC variant panel. A, Schematic of the WT G6PC‐EGFP fusion construct. B, Mean ± SD percentage of G6PC‐EGFP positive transfected cells detected in n = 180 images per variant tested using the Perkin Elmer Operetta System at ×20 magnification. Stats analysis of each individual variant compared to WT with Welch's unpaired two‐tailed *t* test, all unmarked are not significant. C, Representative DAPI stained ×20 magnification images from controls and representative variants with low (Q347X), medium (R83C), and high (Q347X) G6PC‐EGFP expression in mCherry positive transfected cells. DAPI, 4′,6‐diamidino‐2‐phenylindole; WT, wild type

The first step of this study was to quantify overall G6PC content of a panel of variants in a uniform context using a tagged fusion protein construct (Figure 2A). The percent of transfected cells expressing EGFP was calculated for each of the 29 variants and compared to WT (Figure 2B). In this screen variants with premature termination codons are essentially undetectable with the highest being 6.7% (p.Gln27Ter) of WT level, while missense variants display a wide range of values from 29.1% (p.Gln54Pro) to 148.0% (p.Val304Ile) of WT level, with six showing significantly less G6PC‐EGFP amount than WT. Two VUS, p.Val304Ile and p.Val308Ile, showed significantly more G6PC‐EGFP protein level than WT G6PC‐EGFP, while 15 variants had significantly lower protein level. Notably, one of the eight VUS tested, p.Cys109Tyr, showed reduced protein expression at roughly 33.7% WT level. Example images (Figure 2C) are included showing WT (positive) and mock transfected (negative) controls, along with representative low, medium, and high protein level variants.

**FIGURE 3:**
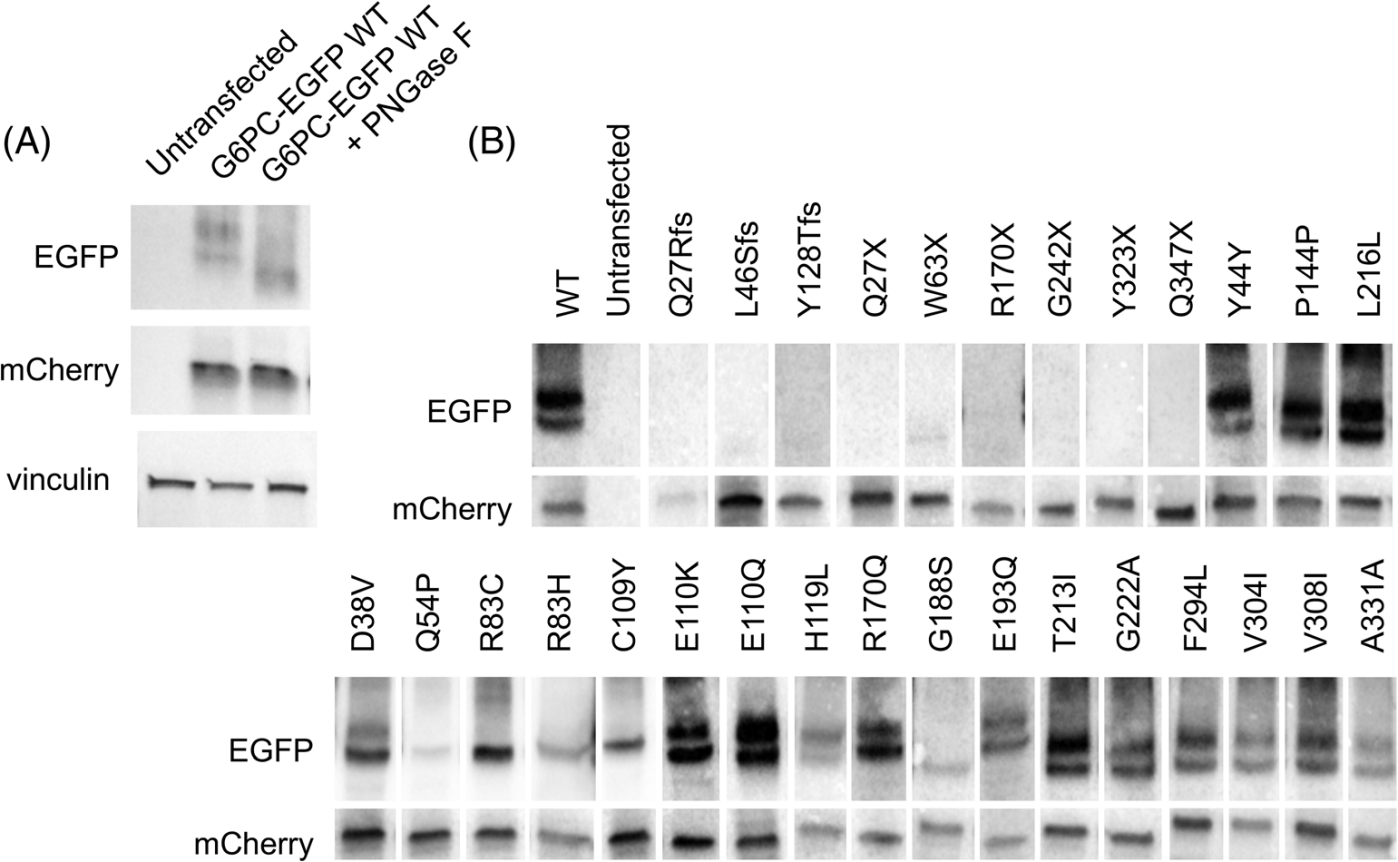
G6PC N‐linked glycosylation is altered in several G6PC variants. G6PC glycosylation status is altered for multiple missense variants of known and uncertain pathogenicity. A, Western blot of whole cell lysate from HepG2 cells transfected with G6PC‐EGFP +/− treatment with PNGase F to de‐glycosylate the protein. B, Representative western blots for all protein expressing variants showing varied patterns of the WT double band pattern. WT, wild type

As G6PC is reported to have N‐linked glycosylation at the N96 position, we next qualitatively examined all variants in the G6PC‐EGFP fusion protein construct by western blot. WT G6PC‐EGFP shows a distinct double band, and when treated with PNGase F to deglycosylate the protein only a single band is seen, indicating the higher molecular weight bands are glycosylated forms of G6PC (Figure 3A). Analysis of the variants revealed the banding pattern is altered in some, likely attributed to some alteration in protein glycosylation (Figure 3B). This confirmed there was little to no detectable G6PC‐EGFP protein for the three frameshift and six nonsense variants tested. The three synonymous variants displayed a two‐band pattern consistent with WT, while the five of the 16 missense variants appear to have only a single band indicative of a glycosylation defect. Notably these five variants with a single band are all among the six variants shown to have significantly reduced protein amount in Figure 2B. The remaining variant with reduced protein amount, p.Asp38Val, did retain a doublet banding pattern; however, the higher molecular weight band is visibly fainter.

**FIGURE 4:**
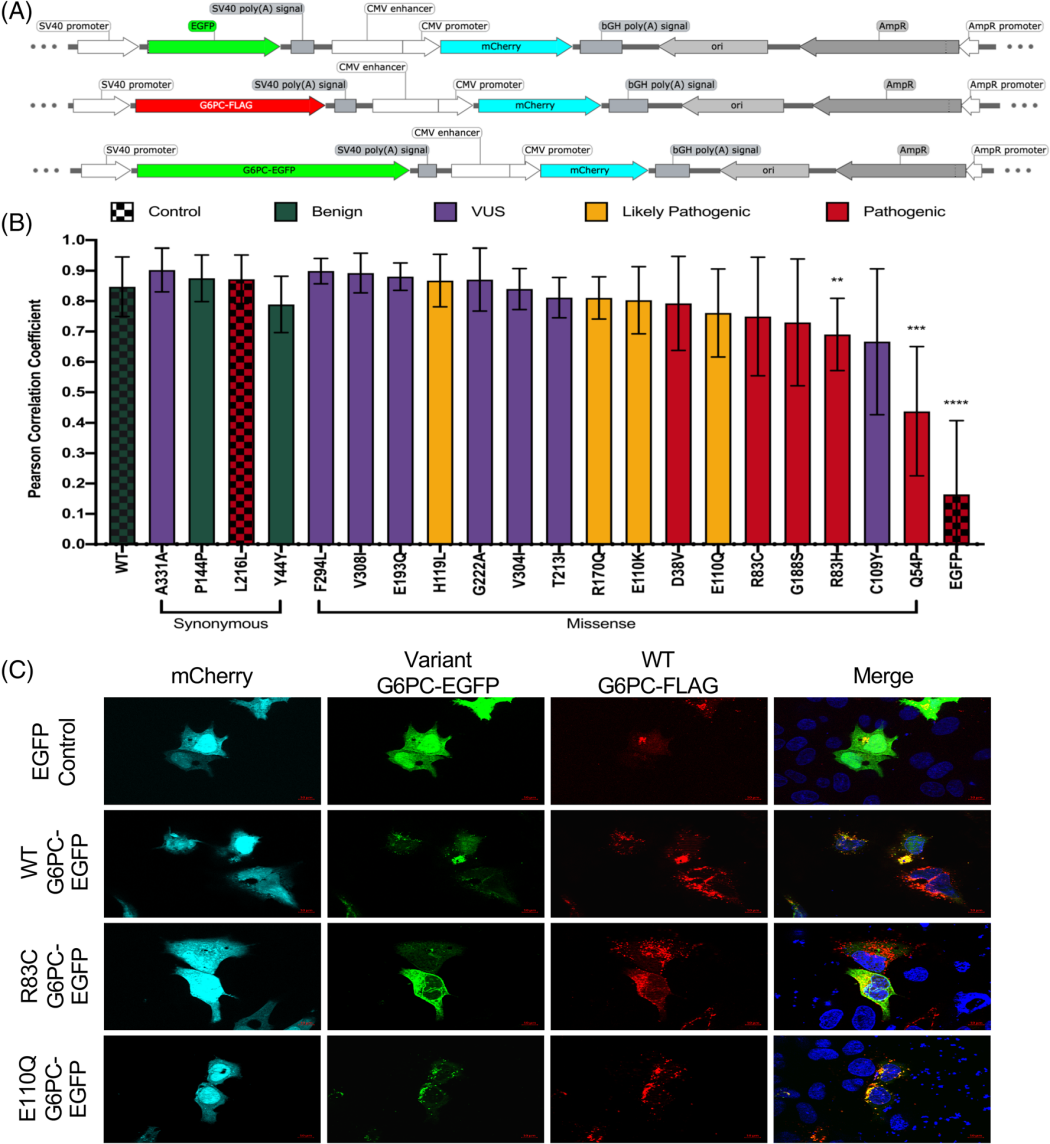
Several G6PC variants have altered protein localization. Abnormal localization may contribute to pathogenicity in some variants. A, Schematic of the fusion constructs with EGFP‐only control plasmid (top), G6PC‐FLAG WT construct (middle), and G6PC‐EGFP construct (bottom) mutagenized with each variant. B, Mean ± SD Pearson correlation coefficient calculated by Zeiss Zen Pro software colocalization module in n = 9 cells imaged at ×63 magnification for each variant. Stats analysis of each individual variant compared to WT with Welch's unpaired two‐tailed *t* test, all unmarked are not significant. C, Representative images showing protein localization compared to WT in controls and two select variants (R83C, Pearson 0.7494; E110Q, Pearson 0.7597). WT, wild type

We next examined intracellular distribution of the G6PC‐EGFP tagged fusion protein. Localization was analyzed using WT G6PC‐FLAG was cotransfected with each protein expressing variant in G6PG‐EGFP (Figure 4A) and an average Pearson colocalization score was plotted (Figure 4B). Cotransfection of two WT constructs had an average Pearson colocalization score of 0.847, while WT G6PC‐FLAG with the EGFP negative control had an average of 0.164. Representative images (Figure 4C) show the diffuse cytoplasmic pattern of EGFP alone, in contrast to the restricted, punctate appearance of WT G6PC localization. Variants p.Arg83Cys and p.Glu110Gln (R83C and E110Q) demonstrate visual differences, where p.Glu110Gln (Pearson 0.761) retains a restricted punctate pattern and colocalizes well with WT, while p.Arg83Cys (Pearson 0.7494) appears more diffuse and abnormal in localization. Variants with a mean Pearson score below that of p.Arg83Cys all showed visible trends of abnormal localization and had a wider variance reflecting some abnormality in the localization of these variants that warrants further analysis.

**FIGURE 5:**
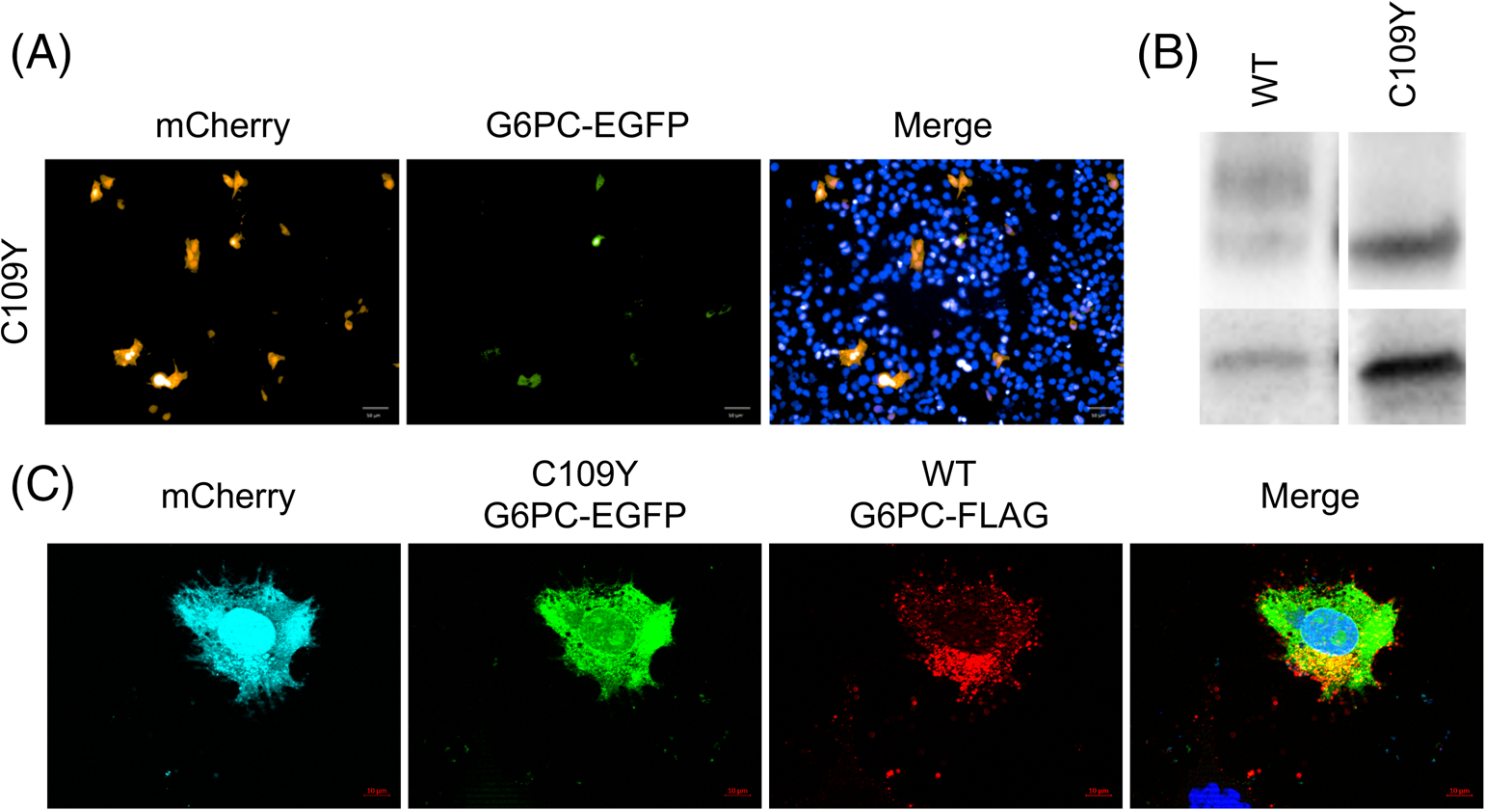
C109Y, a variant of uncertain significance, shows features consistent with pathogenicity. VUS variant C109Y shows features consistent with pathogenicity. A, HepG2 transfected cells showing reduced G6PC‐EGFP protein expression for C109Y (14.85%) compared to WT (44.03%). B, Western blot showing a singular band for C109Y compared to double banding for WT. C, Transfected HepG2 cells showing C109Y localization having a diffuse cytoplasmic pattern compared to a more restricted pattern of WT G6PC‐FLAG expression. VUS, variants of uncertain significance. WT, wild type

Of the eight VUS tested, p.Cys109Tyr was notable for showing abnormal characteristics in each assay and was re‐listed as having conflicting interpretations of pathogenicity in the Clinvar database as of September 2019. In our analysis, high throughput cell imaging showed the protein level was 33.9% of WT (Figure 5A), and only a singular, nonglycosylated band was seen on western blot (Figure 5B). In examining C109Y's localization, it was noted that localization was not aberrant in every cell analyzed. There was much greater intercell variation in localization, where the majority of cells analyzed showed the abnormal diffuse cytoplasmic localization (Figure 5C), and some appeared to retain WT localization patterning. The variation in intracellular distribution, as shown in Figure 4B, may reflect an effect of the variant that is not otherwise quantifiable.

## 4 DISCUSSION

The goal of this study was to systematically examine molecular phenotypes of a panel of G6PC variants, and to apply this methodology to inform about the cause of pathogenicity and the pathogenic potential of VUS. The 29 variants tested provide a representative selection of the types and proportions of those found in the GSD1a patient population and should provide insight into diagnostic ambiguities as well as help guide future “personalized” therapeutic strategies. By examining steady‐state protein levels, glycosylation status, and localization within the cell, we have yielded new information on how each variant affects protein behavior at a molecular level, summarized in Table 1. This information can supplement current databases combining in silico predictions, patient data, and other in vitro reports to give a more comprehensive picture on pathogenicity and its underlying molecular cause for each variant. Using a uniform approach allows us to compare variants and make molecular groupings that could streamline prognostics and therapeutic approvals for like‐variants, as seen in recent FDA approvals for cystic fibrosis medications based on in vitro classification data.^13^

**TABLE 1:** Summary of G6PC variant molecular phenotype results

Protein code	cDNA alteration	Protein alteration	Pathogenicity	Protein level	Localization
EGFP	NA	NA	Control	89.39	0.164
WT	Refseq	None	Control	44.03	0.847
Q27Rfs	c. 79 del C	p.Gln27fs	Pathogenic	4.36****	NA
Q27X	c. 79C>T	p.Gln27Ter	Likely pathogenic	2.94****	NA
D38V	c.113A>T	p.Asp38Val	Pathogenic	20.70****	0.775
Y44Y	c.132C>T	p.Tyr44=	Benign	51.62	0.789
L46Sfs	c.136delC	p.Leu46fs	Likely pathogenic	2.08****	NA
Q54P	c.161 A>C	p.Gln54Pro	Pathogenic	12.81****	0.438***
W63X	c.189 G>A	p.Trp63Ter	Likely pathogenic	1.06****	NA
R83C	c.247C>T	p.Arg83Cys	Pathogenic	22.95****	0.749
R83H	c.248 G>A	p.Arg83His	Pathogenic	28.46***	0.690**
C109Y	c.326 G>A	p.Cys109Tyr	VUS	14.85****	0.666
E110K	c.328 G>A	p.Glu110Lys	Likely pathogenic	47.23	0.803
E110Q	c.328 G>C	p.Glu110Gln	Likely pathogenic	58.39	0.761
H119L	c.356 A>T	p.His119Leu	Likely pathogenic	45.78	0.868
Y128Tfs	c.379_380dupTA	p.Tyr128fs	Likely pathogenic	0.93****	NA
P144P	c.432 G>A	p.Pro114=	Benign	56.40	0.875
R170Q	c.509 G>A	p.Arg170Gln	Likely pathogenic	57.70	0.811
R170X	c.508C>T	p.Arg170Ter	Pathogenic	0.83****	NA
G188S	c.562 G>A	p.Gly188Ser	Pathogenic	24.37****	0.730
E193Q	c.577 G>C	p.Glu193Gln	VUS	53.03	0.881
T213I	c.638 C>T	p.Thr213Ile	VUS	49.35	0.812
L216L	c.648G>T	p.Leu216=	Pathogenic	52.25	0.872
G222A	c.665 G>C	p.Gly222Ala	VUS	60.76	0.871
Q242X	c.724 C>T	p.Gln242Ter	Pathogenic	0.72****	NA
F294L	c.882 C>A	p.Phe294Leu	VUS	55.10	0.899
V304I	c.910 G>A	p.Val304Ile	VUS	65.18*	0.840
V308I	c.922 G>A	p.Val308Ile	VUS	61.97*	0.893
Y323X	c.969 C>A	p.Tyr323Ter	Pathogenic	0.72****	NA
A331A	c.993 G>C	p.Ala331=	VUS	59.92	0.902
Q347X	c.1039C>T	p.Gln347Ter	Pathogenic	1.16****	NA

*Note:* Summary chart of protein level and localization consequences of 29 G6PC variants. Protein level value is the mean percent EGFP positive transfected cells from n = 180 images analyzed at ×20 magnification per variant. Localization value is the mean Pearson correlation coefficient between WT and variant colocalization in n = 9 cells analyzed at ×63 magnification. Significance levels are shown as compared to WT in a Welch's two‐tailed *t* test.

Abbreviation: VUS, variants of uncertain significance, WT, wild type.

^*^ *P* < .05.

^**^ *P* < .01.

^***^ *P* < .001.

^****^ *P* < .0001.

When examining protein levels, these results support the computational prediction that variants causing premature stop codons in disease‐linked genes are almost universally pathogenic, and confirm that the pathogenicity is due to protein absence. Thus, for nonsense and frameshift variants, in silico analysis is likely sufficient and additional in vitro data is not warranted. Generally, the best therapeutic options to address nonsense and frameshift variants would be those that restore protein production, such as gene editing to return to the reference sequence, and gene/mRNA/protein replacement to provide alternate production sources. In the case of GSD1a, protein replacement therapy is complicated by it being a membrane‐bound ER protein. However, advances are being made in clinical trials with gene replacement therapy.^29^, ^30^, ^31^, ^32^, ^33^ Additional therapies for premature stop codon read‐through could be applied to nonsense variants, such as nonsense suppressor tRNA or therapeutic compounds like aminoglycosides.

Our identification of variants with reduced protein amount represents a new molecular phenotype that may be contributing to their pathogenicity. Reduced enzyme quantity could be the underlying cause of lower enzymatic activity. While variants in active site residues (p.Arg83Cys and p.Arg83His) are likely catalytically inactive regardless of protein levels, missense variants with reduced total protein may benefit from therapeutic strategies to increase protein production or decrease degradation.

As previous reports showed substituting the N96 residue to prevent N‐linked glycosylation reduced enzyme activity,^19^ we hypothesized that other variants may alter glycosylation and be an underlying cause of pathogenicity. Our results showed this was a prominent phenotype for missense variants in G6PC and that it warrants examination as a contributor to pathogenicity in glycosylated proteins.

Given that G6PC resides within the ER, we examined localization as a potential contributor to pathogenicity. The abnormal localization is an especially crucial molecular phenotype for a compartmentalized enzyme, where increasing expression or using therapeutics that modulate or enhance function may have severe and unexpected side effects due to its location within cells and altered access to potential substrates.

Interestingly, the missense variants with lower protein level also had abnormal glycosylation and were among the lowest localization scores. The consistent results for six of the missense variants showing significantly decreased protein level, abnormal glycosylation, and trending toward altered localization, suggest a connection among these phenotypes. It is possible each phenotype is distinct, but it is more likely they are linked in a cause/effect fashion. Likely, the altered glycosylation and/or localization within the cells is tagging the protein for degradation, leading to decreased levels. However, the trafficking differences could be due to, or the cause of, abnormal glycosylation. It may be possible for these variants that a singular therapeutic intervention could alleviate all three abnormalities, as such, further investigation is warranted to determine the root cause of the complex molecular phenotype. However, it is currently unknown if these variants in G6PC‐expressing cells of nonhepatic tissues will behave similarly and those studies are currently being conducted.

Finally, in the case of the missense VUS p.Cys109Tyr, this study demonstrates that disease annotation will be an iterative process, requiring continual updates of sequencing and clinical data with in vitro lab analysis to help establish pathogenicity status and its cause for a given variant. When this study began, p.Cys109Tyr was classified as a VUS in ClinVar database, and as it progressed, clinical data emerged for a GSD1a patient homozygous for the p.Cys109Tyr variant, transitioning its status to be likely, but not definitively, pathogenic.^34^ Combined with the data presented here that show this variant, and not a closely linked undetected variant(s), affects function and is consistent with other known pathogenic variants in each assessment performed. We assert that this variant could be conclusively classified pathogenic and provide closure to current and future carriers of the variant on its potential to cause disease.

## CONFLICT OF INTEREST

The authors declare no potential conflict of interest.

## AUTHOR CONTRIBUTIONS

Kathleen L. Plona and Mitchell L. Drumm wrote the manuscript. Kathleen L. Plona designed experiments, acquired, interpreted, and compiled data. Jean F. Eastman provided technical expertise and collected data.

## ETHICS STATEMENT

This article does not contain any studies with human or animal subjects performed by the any of the authors.

## WEB RESOURCES

Clinvar https://www.ncbi.nlm.nih.gov/clinvar/

Ensembl Genome Browser https://uswest.ensembl.org/index.html

Online Mendelian Inheritance of Man http://www.omim.org/

Uniprot https://www.uniprot.org/

VectorBuilder https://en.vectorbuilder.com/
